# Impact of seat position on survival outcomes and anatomically specific severe injury patterns in four-wheeled motor vehicle accidents: a retrospective cohort study at a community emergency department in Japan

**DOI:** 10.1186/s12873-025-01302-z

**Published:** 2025-07-30

**Authors:** Tasuku Uzawa, Yuko Ono, Jun Sugiyama, Kazushi Takayama, Nobuto Nakanishi, Takeyasu Kakamu, Tokiya Ishida, Nozomi Tomita, Kazuaki Shinohara, Joji Kotani

**Affiliations:** 1 Department of Disaster and Emergency Medicine, Graduate School of Medicine, Kobe University, Kobe, Japan; 2 Department of Anesthesiology, Ohta General Hospital Foundation, Ohta Nishinouchi Hospital, Koriyama, Japan; 3 Department of Hygiene and Preventive Medicine, School of Medicine, Fukushima Medical University, Fukushima, Japan

**Keywords:** Driver seat, Front passenger seat, Severe trauma, Traffic accidents, Rear passenger seat

## Abstract

**Background:**

Road traffic accidents are a major healthcare concern worldwide. To improve outcomes for patients injured in motor vehicle crashes, it is crucial to understand the factors associated with mortality and anatomically specific injury severity. Seat position is one of the possible determinants of road traffic injury fatality; however, evidence regarding which seat positions are linked to impaired survival outcomes and anatomically severe injuries remains scarce.

**Methods:**

We conducted a retrospective cohort study of patients injured in four-wheeled vehicle accidents between 2000 and 2022 and admitted to a community teaching hospital in Japan. Seat position was classified as driver seat, front passenger seat, or rear passenger seat. The primary endpoint was in-hospital mortality. Other outcomes included severe trauma, defined as an Injury Severity Score (ISS) of > 15, and anatomically specific severe injuries of the head and neck, chest, abdomen, pelvis, and extremities, defined as an Abbreviated Injury Scale score of ≥ 3.

**Results:**

Among 5,906 eligible patients, 4,104 (69.5%) were driver seat occupants, 1,009 (17.1%) were front passenger seat occupants, and 793 (13.4%) were rear passenger seat occupants. After adjusting for potential confounders such as age, sex, admission year, season, presentation time, presentation day, prehospital length of stay, vehicle configuration, collision type, seatbelt use, airbag deployment, and involvement in high-energy trauma using logistic regression analysis, rear passenger seat occupants had a lower risk of hospital mortality (adjusted odds ratio [AOR], 0.396; 95% confidence interval [CI], 0.216–0.727) and a lower risk of severe trauma with an ISS of > 15 (AOR, 0.428; 95% CI, 0.308–0.596) than driver seat occupants. Additionally, rear seat occupants were less likely to sustain serious injuries to the chest (AOR, 0.474; 95% CI, 0.333–0.673) and abdominal or pelvic contents (AOR, 0.373; 95% CI, 0.218–0.639) than driver seat occupants.

**Conclusion:**

Our results suggest that driver seat occupants require special attention because of their higher risk of adverse outcomes and anatomically severe injuries. These findings will be useful for vehicle occupants, emergency medical professionals, and automobile manufacturers.

**Clinical trial number:**

Not applicable.

**Supplementary Information:**

The online version contains supplementary material available at 10.1186/s12873-025-01302-z.

## Background

Road traffic accidents are a major healthcare concern and place a significant economic burden on society. According to the World Health Organization, approximately 1.19 million road traffic accidents occur annually worldwide [1]. Road traffic accidents are the leading cause of mortality among children and young people aged 5–29 years and the 12th leading cause of death across all ages [1]. Recent estimates suggest that the global economic cost of road traffic injuries is USD 1.8 trillion, equivalent to 10–12% of the global gross domestic product [2]. Therefore, road traffic injuries are a critical healthcare and economic issue that warrants increased attention.

To improve the survival and functional outcomes of injured patients involved in motor vehicle crashes, understanding the factors associated with mortality and anatomically specific injury severity is crucial. Seat position, classified as driver seat, front passenger seat, and rear passenger seat, is a potential determinant of road traffic injury fatality. Compared with the rear passenger seat, the front seat—particularly the driver seat—has several hard components, such as the steering wheel, dashboard, and windscreen. These components may strike the front occupants during a traffic accident and increase the likelihood of more anatomically severe injuries, thereby worsening survival outcomes. However, evidence regarding whether and which seat position is related to anatomically severe injuries and impaired survival outcomes remains scarce, with conflicting results reported in prior studies [3–8]. For instance, two studies proposed that front seat occupants were more likely to experience fatal outcomes than rear seat occupants [4, 5], but these studies did not account for variables beyond seatbelt use, airbag presence, age, and sex. By contrast, two other studies suggested that rear seat occupants faced a higher risk of mortality than did front seat occupants, although their findings were confounded by collision type, seatbelt use, airbag deployment, and the involvement of high-energy injury mechanisms [6, 7]. Thus, whether survival outcomes are affected by seat position remains unclear. Furthermore, prior studies have not adequately addressed whether seat position influences anatomically specific injury severities measured by the Abbreviated Injury Scale (AIS) or Injury Severity Score (ISS), which are critical trauma care parameters [9, 10]. To formulate improved treatment strategies for injured patients, it is essential to understand the differences in organ-specific severities by seat position. Our trauma database prospectively captures these variables, allowing for rigorous statistical adjustments and detailed outcome comparisons.

The goal of the current study was to determine whether and which seat position was associated with impaired survival outcomes and anatomically specific severe injuries among patients injured in four-wheeled motor vehicle accidents. We hypothesized that the rear passenger seat position is associated with decreased mortality and anatomical severity, even after adjusting for potential confounders.

## Methods

### Study design, setting, and participants

This retrospective cohort study was conducted at a community teaching hospital in a Japanese city located approximately 200 km northeast of Tokyo. The study protocol was approved by the ethics committee at Ohta Nishinouchi Hospital on 19 July 2024 (approval no. 9_2024). The committee waived the requirement for patient consent because of the observational nature of the study, which involved no interventions and focused solely on outcomes from routine trauma practice. Opt-out information was provided on the hospital’s website (https://www.ohta-hp.or.jp/nishi/about/attempt/clinical_research). The study hospital serves as the only tertiary and referral emergency medical center within a 50-km radius, catering to approximately 500,000 inhabitants. Annually, the emergency department (ED) receives more than 5,500 ambulances, with approximately 25% of these cases involving trauma patients. Detailed information about the study facility, including its hospital-based physician-staffed ambulance, staffing levels, emergency ward bed capacity, number of operating rooms, and catheterization laboratory availability for trauma patients, has been reported previously [11–13].

The study enrolled all injured patients involved in four-wheeled motor vehicle crashes who were admitted to the study ED between 1 January 2000 and 31 December 2022. Patients injured in accidents involving other types of vehicles, such as pedestrians and motorcycle, scooter, or bicycle occupants, were excluded. Patients injured in truck-type vehicle accidents were also excluded because these vehicles lack rear passenger seats. Additionally, victims of other vehicle accidents, such as those involving buses or agricultural tractors, were excluded. Patients with missing data were excluded; only complete data sets were used for all relevant analyses. The retrospective nature of the study predetermined the sample size, making an a priori estimation of statistical power impossible. Thus, the analysis was conducted on all collectable data from the study period. The minimal anonymized data set used in this study is available as S1 Data in the supplementary materials.

### Exposures and outcome measurement

The main exposure in this study was seat position, classified as driver seat, front passenger seat, and rear passenger seat. The primary outcome measure was in-hospital mortality among these groups. Other outcomes of interest were anatomically specific severe injuries, defined as those with an AIS score of ≥ 3 for each anatomical site, and severe traumatic injury, defined as an ISS of > 15. These definitions and measurements of severe trauma have been used in previous studies [14–17]. All trauma parameters were assessed by a single author (K.S.), a board-certified emergency physician specializing in trauma care, who entered the data into the database at his earliest convenience. To minimize bias, the author responsible for scoring trauma-related outcomes did not participate in planning or performing the statistical analyses.

### Covariates

Covariates potentially associated with seat position and outcomes [11–23] were extracted from the hospital-based electronic medical database. We collected patient demographics (age and sex), ED admission year (2000–2007, 2008–2015, or 2016–2022), season (spring: March–May, summer: June–August, autumn: September–November, or winter: December–February), ED presentation time (8:00–16:59, 17:00–23:59, or 0:00–7:59), ED presentation day (weekday or weekend), prehospital length of stay, and vehicle factors such as seatbelt use (belted, unbelted, or improper seatbelt use), airbag deployment (equipped and deployed, equipped but not deployed, or not equipped), vehicle configurations (standard vehicle or K-car vehicle), collision type (frontal collision, lateral collision, rear-end collision, rollover collision, complex collision, or other type of collision), and high-energy trauma as defined in the Japan Advanced Trauma Evaluation and Care guideline (e.g., death in the same compartment or ejection from the automobile) [23]. Vehicle configuration was dichotomized into standard vehicles and K-car vehicles. K-car vehicles, also known as “mini vehicles,” comprise the smallest category of Japanese expressway-legal motor vehicles. A K-car vehicle was defined as one having an engine displacement of < 660 cc, vehicle length of < 3.4 m, width of < 1.48 m, and height of < 2.0 m according to the Japanese Road Transport Vehicle Act [24]. The vehicle configuration was defined as a standard vehicle if any of these criteria were exceeded. Complex collisions were defined as traffic accidents involving multiple mechanisms of injury—for example, a chain-reaction crash involving both frontal and rear-end collisions, or a frontal collision with an oncoming vehicle following an initial lateral impact. “Other” types of collisions referred to traffic accidents with additional elements, such as a vehicle becoming stuck in a groove after a frontal collision or falling into a rice paddy following a lateral impact. Traffic accident details—including seat position, seatbelt use, airbag deployment, vehicle configuration, collision type, and high-energy trauma—were initially extracted from care reports written by emergency medical services personnel. When data were missing, unclear, or ambiguous, one of the investigators (author K.S.) conducted interviews with the involved emergency medical services personnel, the patients themselves, or co-passengers to clarify the details. These post hoc interviews were conducted within a few days of the accident. If traffic accident details remained uncertain despite these efforts, the data were recorded as missing and excluded from analysis. To assess the potential for selection bias, we compared the characteristics of patients included in the analysis with those excluded because of missing accident-related details.

### Statistical analysis

All statistical analyses were planned *a priori*. Baseline clinical characteristics among the study participants were initially assessed. Differences in continuous variables across the three groups were compared using one-way analysis of variance after verifying the normal distribution of the data with the Kolmogorov–Smirnov test. If the data were not normally distributed, the Kruskal–Wallis test was used instead. Differences in categorical variables among the three groups were compared using the chi-squared test. Logistic regression was employed to calculate crude odds ratios and adjusted odds ratios (AORs) for in-hospital mortality and anatomically specific severe injuries (AIS score of ≥ 3). A set of potential confounders was chosen a priori based on previous findings [11–23] and biological plausibility. These selected variables were age, sex, admission year, season, presentation time, presentation day, prehospital length of stay, vehicle configuration, collision type, seatbelt use, airbag deployment, and involvement in high-energy trauma. All categorical variables were dummy-coded and included as explanatory variables in the multiple logistic regression model, while age was treated as a continuous variable. We tested for effect modification between rear passenger seat occupants and older patients (aged > 65 years), as well as between rear passenger seat and standard vehicle occupants, by including their interaction terms in a logistic regression model with in-hospital mortality as the dependent variable. The results did not indicate effect modification (data not shown); therefore, interaction terms were not included in the final model. We omitted an AIS score for the face of ≥ 3 from the multivariable analysis because of the relatively low number of events, which was insufficient for inclusion in a multivariable logistic regression model adjusting for the number of confounders described above. Multicollinearity was assessed using the variance inflation factor. The Hosmer–Lemeshow test and the c-statistic were used to evaluate the model’s goodness of fit and discrimination ability, respectively. The c-statistic is a measure commonly used in medical research to assess the performance of predictive models, particularly logistic regression. A c-statistic of 0.5 indicates no predictive power (equivalent to random guessing), whereas a value closer to 1.0 reflects strong predictive performance. Statistical analyses were performed using IBM SPSS Statistics version 29.0 (IBM Corp., Armonk, NY, USA). A type I error rate of *p* < 0.025 was used when comparing outcomes between driver seat occupants and front or rear seat occupants, based on Bonferroni adjustment for multiple comparisons. Otherwise, a conventional *p*-value of < 0.05 was considered statistically significant.

### Subanalysis

To assess the robustness of our primary analysis, a sensitivity analysis using a different definition of the exposure was conducted. The multivariable analysis comparing rear seat occupants versus front seat occupants (i.e., driver and front passenger seat occupants combined) was repeated for primary outcome, with the rear seat position set as the reference. To explore how sex differences may influence in-hospital mortality and injury patterns, logistic regression analysis was also conducted separately for female and male subgroups. Finally, we examined factors associated with in-hospital mortality specifically among rear passenger seat occupants. For this analysis, a univariable logistic regression model was used for each covariate to calculate crude odds ratios for in-hospital mortality within this group.

## Results

### Participant flow

**Fig. 1:**
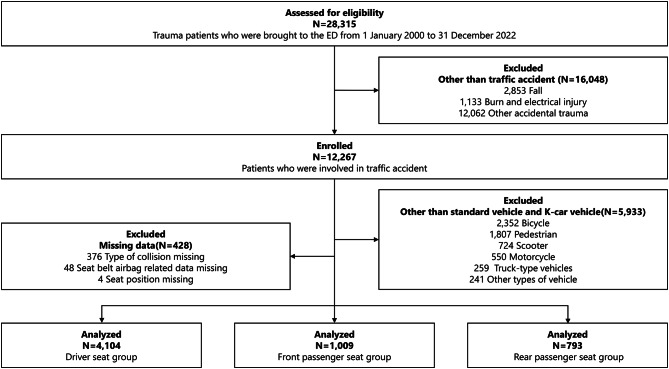
Participant flow chart. ED, emergency department

During the study period, 28,315 trauma patients were transported to the study facility, of whom 12,267 (43.3%) had been involved in traffic accidents (Fig. 1). Among these, 5,933 patients injured in accidents involving vehicles other than standard or K-car vehicles and 428 patients with missing data were excluded from the analysis. The amount of missing data was small (< 3.5%) for all relevant analyses. The crude analysis included the remaining 5,906 patients with complete data sets. Of these, 4,104 (69.5%) were classified into the driver seat group, 1,009 (17.1%) into the front passenger seat group, and 793 (13.4%) into the rear passenger seat group (Fig. 1). The differences in characteristics between patients included in the analysis and those excluded because of missing traffic accident details are presented in S1 Table.

### Patient characteristics

Patient characteristics according to seat positions are summarized in Table 1. Significant imbalances were observed among the three groups in terms of age, sex, ED admission year, ED presentation day, prehospital length of stay, vehicle configuration, collision type, seatbelt use, airbag use, and high-energy trauma. These variables, along with season and ED presentation time, were included in the multivariable logistic regression model.

**Table 1:** Demographic and clinical characteristics of study participants according to seat position

	Seat position			
	Driver seat (*n* = 4,104)	Front passenger seat (*n* = 1,009)	Rear passenger seat (*n* = 793)	*p*-value
**Age, years**				
Median (IQR)	42 (27–58)	36 (19–63)	25 (10–56)	**< 0.001**
**Sex**				
Male	2,378 (57.9)	339 (33.6)	320 (40.4)	**< 0.001**
Female	1,726 (42.1)	670 (66.4)	473 (59.6)	
**ED admission year**				
2000–2007	1,969 (48.0)	547 (54.2)	409 (51.6)	**0.004**
2008–2015	1,334 (32.5)	302 (29.9)	245 (30.9)	
2016–2022	801 (19.5)	160 (15.9)	139 (17.5)	
**Season**				
Spring (March–May)	987 (24.0)	249 (24.7)	180 (22.7)	0.704
Summer (June–August)	1,102 (26.9)	257 (25.5)	213 (26.9)	
Autumn (September–November)	1,052 (25.6)	261 (25.9)	193 (24.3)	
Winter (December–February)	963 (23.5)	242 (24.0)	207 (26.1)	
**ED presentation time**				
8:00–16:59	2,156 (52.5)	542 (53.7)	427 (53.8)	0.237
17:00–23:59	1,213 (29.6)	312 (30.9)	243 (30.6)	
0:00–7:59	735 (17.9)	155 (15.4)	123 (15.5)	
**ED presentation day**				
Weekday	3,000 (73.1)	658 (65.2)	474 (59.8)	**< 0.001**
Weekend	1,104 (26.9)	351 (34.8)	319 (40.2)	
**Prehospital length of stay**,** minutes**				
Median (IQR)	41 (29–58)	42 (29–59)	45 (29–66)	**< 0.001**
**Vehicle configuration**				
K-car vehicles	2,155 (52.5)	609 (60.4)	588 (74.1)	**< 0.001**
Standard vehicles	1,949 (47.5)	400 (39.6)	205 (25.9)	
**Collision type**				
Frontal collision	2,137 (52.1)	507 (50.2)	314 (39.6)	**< 0.001**
Lateral collision	554 (13.5)	178 (17.6)	155 (19.5)	
Rear-end collision	386 (9.4)	95 (9.4)	95 (12.0)	
Rollover collision	502 (12.2)	96 (9.5)	93 (11.7)	
Complex collision	454 (11.1)	107 (10.6)	104 (13.1)	
Other type of collision	71 (1.7)	26 (2.6)	32 (4.0)	
**Seat belt**				
Unbelted	725 (17.7)	272 (27.0)	628 (79.2)	**< 0.001**
Belted	3,339 (81.4)	687 (68.1)	132 (16.6)	
Improper seatbelt use	40 (1.0)	50 (5.0)	33 (4.2)	
**Airbag**				
Not equipped	1,414 (34.5)	420 (41.6)	785 (99.0)	**< 0.001**
Equipped but not deployed	1,299 (31.7)	320 (31.7)	4 (0.5)	
Equipped and deployed	1,391 (33.9)	269 (26.7)	4 (0.5)	
**High-energy trauma**				
Yes	1,647 (40.1)	340 (33.7)	283 (35.7)	**< 0.001**
No	2,457 (59.9)	669 (66.3)	510 (64.3)	

Categorical variables are expressed as n (%), and continuous variables are expressed as median (IQR)

IQR, interquartile range; ED, emergency department

### Primary outcome

**Fig. 2:**
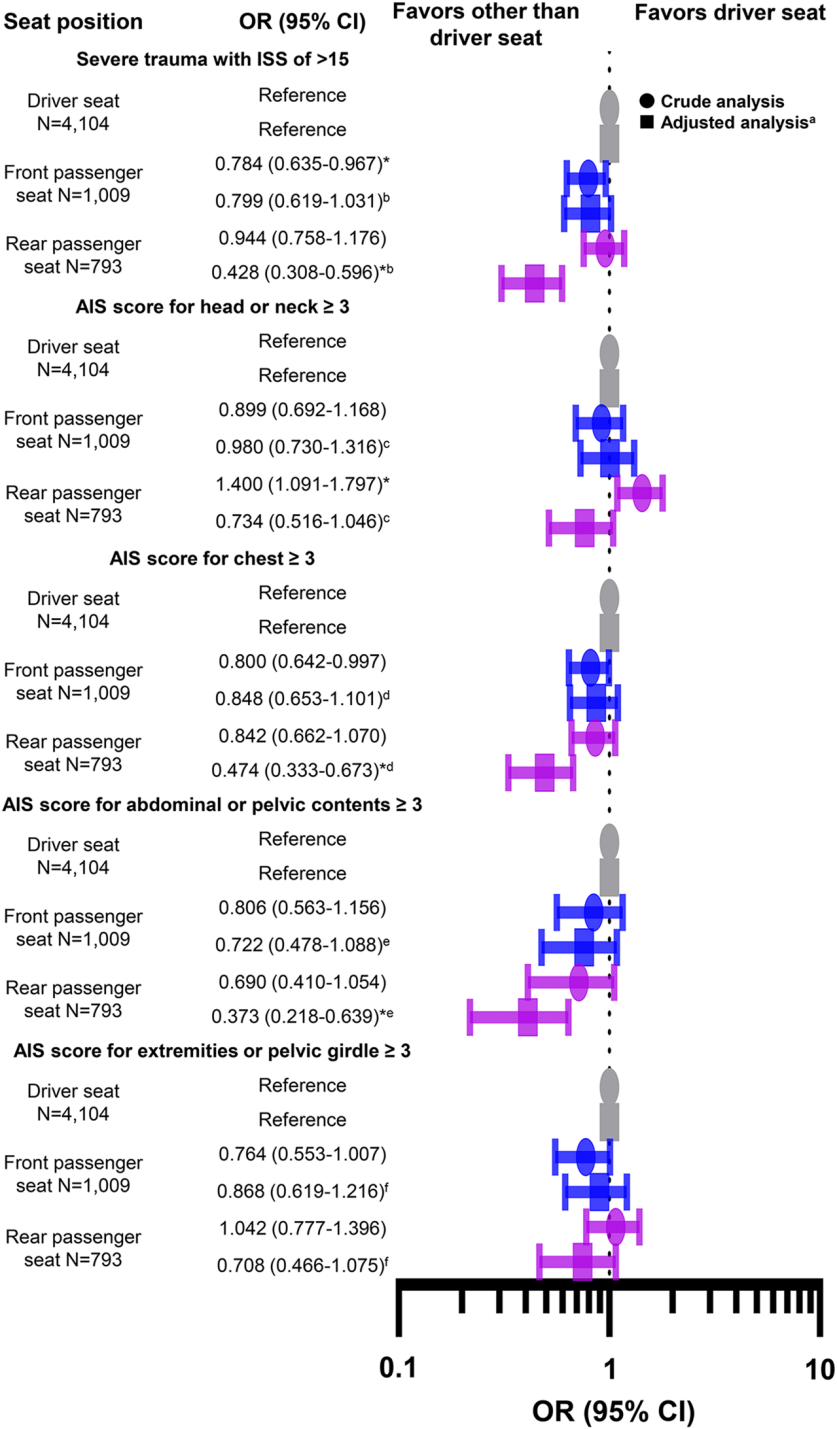
Odds ratios for in-hospital mortality among study participants. The reference group is the driver seat occupants. In the multivariable analysis, the rear passenger seat position was associated with a lower risk of mortality than the driver seat position. ^a^Adjustment for potential confounders: age, sex, admission year, season, presentation time, presentation day, prehospital length of stay, vehicle configuration, collision type, seatbelt use, airbag deployment, and involvement in high-energy trauma. ^b^Good model fit was verified by the Hosmer–Lemeshow test (*p* = 0.252); the c-statistic for the model was 0.880. OR, odds ratio; CI, confidence interval. *p < 0.025

The odds ratios for in-hospital mortality among the driver seat, front passenger seat, and rear passenger seat groups are presented in Fig. 2 and S2 Table. The multivariable analysis revealed that the rear passenger seat position was associated with a lower risk of mortality than the driver seat position (AOR, 0.396; 95% confidence interval [CI], 0.216–0.727).

### Other outcomes

**Fig. 3:**
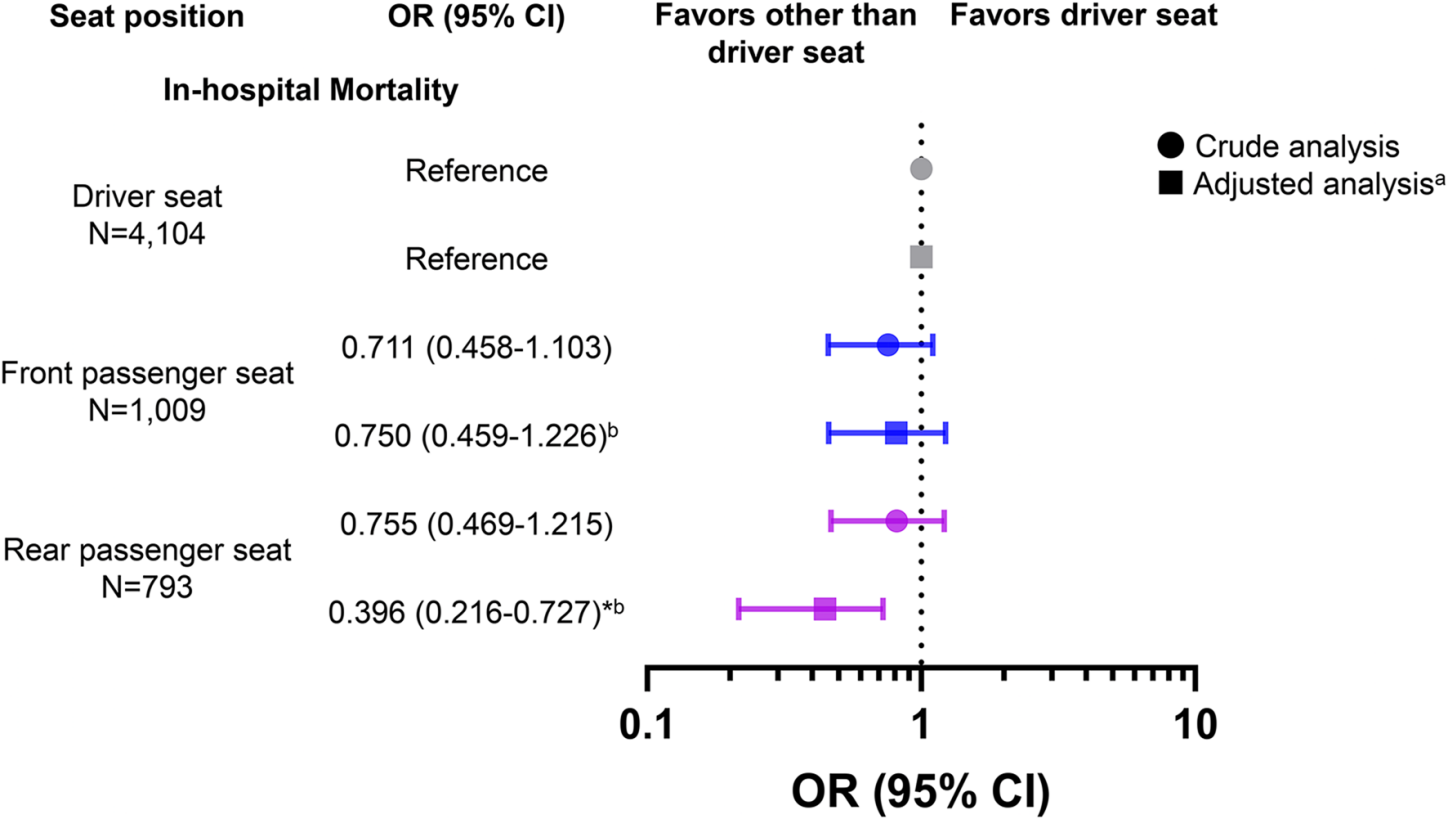
Odds ratios for severe trauma with ISS of > 15 and anatomical site-specific severe injury with AIS score of ≥ 3 for each body component among study participants. The reference group is the driver seat occupants. In the multivariable analysis, the rear passenger seat group exhibited a lower risk of severe trauma with an ISS of > 15 and severe injuries to the chest and abdominal or pelvic contents than the driver seat group. ^a^Adjustment for potential confounders: age, sex, admission year, season, presentation time, presentation day, prehospital length of stay, vehicle configuration, collision type, seatbelt use, airbag deployment, and involvement in high-energy trauma. ^b^Good model fit was verified by the Hosmer–Lemeshow test (*p* = 0.101); the c-statistic for the model was 0.860. ^c^Good model fit was verified by the Hosmer–Lemeshow test (*p* = 0.184); the c-statistic for the model was 0.813. ^d^Good model fit was verified by the Hosmer–Lemeshow test (*p* = 0.107); the c-statistic for the model was 0.851. ^e^Good model fit was verified by the Hosmer–Lemeshow test (*p* = 0.275); the c-statistic for the model was 0.848. ^f^Good model fit was verified by the Hosmer–Lemeshow test (*p* = 0.598); the c-statistic for the model was 0.847. AIS, Abbreviated Injury Scale; ISS, Injury Severity Score; OR, odds ratio; CI, confidence interval. *p < 0.025

Associations between seat positions and anatomically specific severe injuries are shown in Fig. 3 and S3 Table. After adjusting for the potential confounders, the rear passenger seat group exhibited a lower risk of severe trauma with an ISS of > 15 (AOR, 0.428; 95% CI, 0.308–0.596), as well as a lower risk of severe injuries to the chest (AOR, 0.474; 95% CI, 0.333–0.673) and abdominal or pelvic contents (AOR, 0.373; 95% CI, 0.218–0.639), than the driver seat group.

### Subanalysis

In the sensitivity analyses, the adjusted association of decreased in-hospital mortality among rear seat occupants persisted when using different definitions for exposure (S1 Fig.). In the female subgroup, multivariable analysis showed that the rear passenger seat position was associated with a lower risk of mortality than the driver seat position. A similar trend was observed in the male subgroup, although the difference did not reach statistical significance (S2 Fig.). Additionally, the adjusted association of a decreased risk of severe trauma with an ISS of > 15 and severe chest injuries among rear seat occupants was observed regardless of sex differences (S3 Fig.). Notably, among rear passenger seat occupants, all injured patients who wore a seatbelt survived. The proportion of unbelted patients among rear passenger occupants was high (79.2%, 628 of 793), with a 2.7% mortality rate (17 of 628). Improper seatbelt use was also associated with increased in-hospital mortality (S4 Table).

## Discussion

This retrospective cohort study at a community teaching hospital in Japan revealed that occupants of the rear passenger seat position had decreased hospital mortality and a lower risk of severe trauma with an ISS of > 15 compared with occupants of the driver seat position. Additionally, rear passenger seat occupants were less likely to sustain serious injuries to the chest and abdominal or pelvic contents. These findings suggest that driver seat occupants require special attention because of their higher risk of adverse outcomes and anatomically severe injuries.

Several potential mechanistic explanations could account for the more serious injuries to the chest and abdominal and pelvic contents observed among driver seat occupants than among rear passenger seat occupants. First, the driver seat has several hard components, such as the steering wheel, dashboard, and windscreen, which are absent or less prominent in the rear passenger seat position. Driver seat occupants are more likely to come into contact with these components during a collision. Additionally, when an airbag deploys without proper seatbelt use, the resulting large punchout and membrane forces can cause severe chest, neck, and facial injuries to drivers [22]. Previous studies have also suggested that the steering wheel can contribute to severe thoracic and abdominal injuries in drivers during collisions [25]. Our findings align with these previous observations [22, 25]. Our subgroup analysis also showed that the increased risk of severe trauma with an ISS of > 15 and severe injuries to the chest among driver seat occupants, compared with rear passenger seat occupants, persisted regardless of sex differences. These findings collectively highlight the importance of developing safer restraint systems for drivers. An earlier experimental study demonstrated that an enhanced safety system—incorporating features such as a seatbelt force limiter, pretensioner, and energy-absorbing steering column—effectively reduced injury risks in simulated scenarios involving four-wheeled vehicle drivers [26]. More severe anatomical injuries can lead to poorer survival outcomes, as demonstrated in this study. These findings may encourage vehicle occupants and automobile manufacturers to prioritize objective safety considerations regarding seat positions and their associated risks in traffic accidents.

Unlike our observations, one previous study showed that after classifying participants according to seat position, the rear passenger seat position was associated with higher mortality than the driver seat position [6]. Similarly, another study indicated that rear seat passengers were at greater risk for mortality and traumatic brain injuries than were front seat passengers and drivers [7]. Several plausible explanations exist for these discrepancies. In these previous studies, differences in seatbelt use, airbag deployment, type of collision, vehicle configuration, and involvement of high-energy injury mechanisms were not considered when comparing survival outcomes and injury severity between the rear passenger and driver groups. Seatbelt use is known to greatly reduce motor vehicle occupant fatalities [21], but rear-seat passengers are less likely to use such restraints [3, 27]. Failure to adjust for seatbelt use in previous studies could have contributed to the observed increased mortality in the rear passenger group. In agreement with previous findings [3, 27], this study showed that the proportion of unbelted patients among rear passenger occupants was high, and both unbelted and improper seatbelt use were associated with an increased risk of in-hospital mortality. In our study, the most common type of collision was frontal collision, during which unbelted rear seat passengers are likely to move forward, making them prone to severe injuries from collisions with the instrument panel or windshield glass and, in some cases, fatal injuries due to ejection from the vehicle. These findings highlight the need to educate passengers about the importance of wearing a seatbelt when riding in the rear seat [28]. Based on our results, we propose public health campaigns to raise awareness about seatbelt use among rear seat passengers. Our findings also present an opportunity for policymakers to consider implementing stronger rear seat belt legislation to improve occupant safety.

Another study compared the risks of anatomically severe injuries (AIS score of ≥ 3) to any body region between belted front and rear seat occupants. Unlike our findings, the study suggested that the rear seat posed a higher risk of anatomically severe injuries for adults over 16 years of age [8]. Several plausible explanations exist for these discrepancies as well. First, in the previous study, differences other than seatbelt use, collision type, age, and sex were not considered, whereas our study adjusted for additional important confounders such as airbag deployment, time of presentation, day of presentation, prehospital length of stay, vehicle configuration, and high-energy trauma. The interaction of these unmeasured confounders may partially explain the differences between our findings and those of the previous study. Furthermore, differences in outcome measurements, patient populations, medical settings, and healthcare systems between our study and previous studies [6–8] may also contribute to the discrepancies. We believe that some combination of these factors likely accounts for the differences between our findings and those of previous research.

Our findings align to some extent with those of previous studies. For instance, one report found that the rear seat position was associated with decreased mortality and severe injury, even after adjusting for potential confounding factors such as age, sex, seatbelt use, and airbag presence [5]. Similar trends were observed in children under 15 years of age in different settings [4]. Our data corroborate these findings through analyses adjusted for the aforementioned important confounding factors, albeit in a different patient population, geographical region, outcome measurement, and healthcare system than in previous studies. Additionally, we believe our study offers significant advantages. Previous studies classified seatbelt status as simply belted or unbelted and airbag status as either deployed or not deployed [4, 5]. By contrast, we categorized the seatbelt status as belted, unbelted, and improper seatbelt use and the airbag status as equipped and deployed, equipped but not deployed, and not equipped. Given that both proper seatbelt use and airbag deployment are critical in preventing severe injury and death [19, 21, 22], our detailed classifications and their inclusion in the multivariable analysis are particularly important. Taken together, our results, alongside previous findings, collectively delineate the characteristic differences in injury types, outcomes, and required medical resources among traffic accident victims based on seat positions.

We believe our findings have several important implications for consumers, medical personnel, and automobile manufacturers. First, our study provides valuable safety information for consumers. Highlighting the relatively lower risks associated with the rear passenger seat position compared with the driver seat could influence consumers’ seat position choices. For example, when driving a four-wheeled vehicle, placing passengers in the rear seat may enhance their safety. Second, our findings could assist prehospital medical personnel in patient management. If a traffic accident victim is a driver, prehospital teams can anticipate the possibility of severe internal injuries (e.g., pelvic, abdominal, or chest trauma), even when external injuries appear minor. In our study, these findings were consistent regardless of sex differences. This knowledge could help prioritize the transfer of drivers to tertiary medical centers. Similarly, hospital emergency teams could use this information to prepare for emergency interventions such as endotracheal intubation and surgery when treating injured drivers. Third, our findings should encourage automobile manufacturers to invest in developing enhanced safety technologies to further reduce risks associated with different seat positions.

### Limitations and strengths

The current study has several limitations. First, as a single-site observational study, the generalizability of the findings may be limited. Similar studies conducted in other settings with different road safety laws and vehicle safety standards may yield different results. For example, the rate of proper seatbelt use among rear passenger seat occupants in this study was low and suboptimal, which may have biased our findings toward the null hypothesis. In settings with higher safety awareness and better seatbelt compliance among rear seat occupants, the observed benefits associated with the rear passenger seat position might be even more pronounced. In addition, our study was conducted at a tertiary emergency medical facility in Japan, where patients with severe injuries, but not those with minor injuries, are more likely to be transferred. Consequently, the patient sample in this study may be skewed toward higher injury severity. However, we believe that the associations between seat position and measured outcomes are unlikely to be significantly biased because the selection of patients transported to our hospital was based on injury severity rather than seat position. Similarly, we believe that the Japanese community hospital setting does not strongly bias the observed relationships between seat position, poorer survival outcomes, and increased anatomically specific injury severities.

Second, although we made rigorous adjustments using a logistic regression model, unmeasured factors may have confounded our results, as is common in observational studies. For example, several important covariates, such as vehicle velocity, vehicle model year, social status, insurance status, vasopressor use, and alcohol use at the time of injury [8, 16, 29–32], were not recorded in our database. Additionally, environmental factors such as rain, snow, road surface conditions (e.g., wet, snow-covered, or icy), and fog—which can influence injury severity [33, 34]—were not captured in our dataset. Other unmeasured variables included driver-related factors such as fatigue, drowsiness, underlying health conditions, medication use, fainting, and aggressive driving behavior; roadway characteristics such as lighting conditions, number of lanes, and traffic volume; and temporal aspects like periods of traffic congestion [33–37]. Our study was unable to account for these variables because of the lack of prior data collection. While these factors are potential predictors of severe injuries, they were not directly related to seat position in this study. For instance, these variables do not logically influence whether an occupant is seated in the driver seat, front passenger seat, or rear passenger seat. Therefore, according to the classical epidemiologic framework, these factors do not necessarily meet the criteria for confounding in this study. Further research is needed to determine whether adjusting for these variables would affect the associations between seat position and injury severity observed in this study.

Third, as with any observational study, ours is not without risks of data integrity, validity, and ascertainment bias. Nevertheless, all parameters in this study were prospectively scored and entered into the database by a single board-certified emergency physician specializing in trauma care (author K.S.). In cases where data were missing, questionable, or ambiguous, K.S. interviewed the involved healthcare professionals or patients to clarify the traffic accident details. We believe these quality control efforts helped minimize potential sources of bias.

Fourth, because trauma patients with vital organ injuries can die before arriving at the hospital, there is a potential risk of survivorship bias. However, in Japan, emergency medical personnel are not permitted to pronounce death at the prehospital scene except in cases of obvious death, such as decapitation or complete trunk separation. As a result, most patients with traumatic cardiopulmonary arrest are transferred to the emergency department. Because our study did not exclude patients who were receiving ongoing cardiopulmonary resuscitation upon arrival, we believe the magnitude of this bias is likely to be small.

Despite these limitations, we believe the current study has several strengths. First, our database captures relevant trauma parameters, including anatomically specific injury severities of each body component. These outcomes, which are directly relevant to trauma practice, have not been comprehensively assessed in previous studies. Second, the amount of missing data was small (< 3.5%) across all analyses, minimizing selection bias and enhancing the quality of the multivariable logistic regression analysis. The missing data were not directly related to the main exposure or outcome measurements in this study and were therefore likely to be “missing at random.” Additionally, to assess the potential magnitude of selection bias, we compared the characteristic differences between patients with complete datasets and those with missing traffic accident details (S1 Table). Finally, to mitigate the risk of bias, the investigator who entered the data into the database did not participate in the statistical analyses. Thus, we believe the current study accurately illustrates the impact of seat positions on mortality and anatomically specific injury severity among patients involved in traffic accidents.

## Conclusions

In this retrospective observational study, we found that the rear passenger seat position was associated with lower mortality than the driver seat among patients injured in four-wheeled motor vehicle accidents. We also observed that rear passenger seat occupants were less likely to sustain severe injuries to the chest and abdomen and had a lower risk of severe trauma with an ISS of > 15 than driver seat occupants. We hope these findings will assist vehicle occupants, emergency medical professionals, and automobile manufacturers in considering objective safety factors related to injury in traffic accidents.

## Supplementary Information

Below is the link to the electronic supplementary material.

Supplementary Material 1

Supplementary Material 2: Odds ratios for in-hospital mortality among study participants: front seat occupants versus rear passenger seat occupants. The reference group is the rear passenger seat occupants. The front seat occupants include both the driver seat occupant and front passenger seat occupant. In the multivariable analysis, the front seat position was associated with a higher risk of in-hospital mortality than the rear seat position. ^a^Adjusted for age, sex, admission year, season, presentation time, presentation day, prehospital length of stay, vehicle configuration, collision type, seatbelt use, airbag deployment, and involvement in high-energy trauma. ^b^Good model fit was verified by the Hosmer–Lemeshow test (*p* = 0.606). The c-statistic for the model was 0.880. OR, odds ratio; CI, confidence interval.

Supplementary Material 3: Differences in characteristics between patients who were included in the analysis (complete dataset) and those who were excluded from the analysis because of missing data on traffic accident details.

Supplementary Material 4: Odds ratios for in-hospital mortality among (A) male and (B) female subgroups. The reference group is the driver seat occupants. In the female subgroup, the multivariable analysis showed that the rear passenger seat position was associated with a lower risk of mortality than the driver seat position. A similar trend was observed in the male subgroup, although the difference did not reach statistical significance. ^a^Adjusted for age, sex, admission year, season, presentation time, presentation day, prehospital length of stay, vehicle configuration, collision type, seatbelt use, airbag deployment, and involvement in high-energy trauma. ^b^Good model fit was verified by the Hosmer–Lemeshow test (*p* = 0.493); the c-statistic for the model was 0.857. ^c^Good model fit was verified by the Hosmer–Lemeshow test (*p* = 0.828); the c-statistic for the model was 0.918. OR, odds ratio; CI, confidence interval. *p < 0.025.

Supplementary Material 5: In-hospital mortality among study participants.

Supplementary Material 6: Odds ratios of severe trauma (ISS of > 15) and anatomical site-specific severe injury (AIS score of ≥ 3) for each body component among (A) male and (B) female subgroups. The reference group is the driver seat occupants. An adjusted association of decreased risk of severe trauma (ISS of > 15) and severe chest injuries was observed regardless of sex differences. ^a^Adjusted for age, sex, admission year, season, presentation time, presentation day, prehospital length of stay, vehicle configuration, collision type, seatbelt use, airbag deployment, and involvement in high-energy trauma. ^b^Good model fit was verified by the Hosmer–Lemeshow test (*p* = 0.365); the c-statistic for the model was 0.795. ^c^Good model fit was verified by the Hosmer–Lemeshow test (*p* = 0.361); the c-statistic for the model was 0.888. ^d^Good model fit was verified by the Hosmer–Lemeshow test (*p* = 0.661); the c-statistic for the model was 0.841. ^e^Good model fit was verified by the Hosmer–Lemeshow test (*p* = 0.478); the c-statistic for the model was 0.855. ^f^Good model fit was verified by the Hosmer–Lemeshow test (*p* = 0.455); the c-statistic for the model was 0.838. ^g^The Hosmer–Lemeshow test *p*-value was < 0.001; the c-statistic for the model was 0.871. ^h^Good model fit was verified by the Hosmer–Lemeshow test (*p* = 0.395); the c-statistic for the model was 0.818. ^i^Good model fit was verified by the Hosmer–Lemeshow test (*p* = 0.763); the c-statistic for the model was 0.861. ^j^Good model fit was verified by the Hosmer–Lemeshow test (*p* = 0.742); the c-statistic for the model was 0.851. ^k^Good model fit was verified by the Hosmer–Lemeshow test (*p* = 0.785). AIS, Abbreviated Injury Scale; ISS, Injury Severity Score; OR, odds ratio; CI, confidence interval. *p < 0.025.

Supplementary Material 7: Severe trauma with ISS of > 15 and anatomical site-specific severe injury with AIS score of ≥ 3 for each body component among study participants.

Supplementary Material 8: Factors associated with in-hospital mortality among rear passenger seat occupants.

## Data Availability

The minimal anonymous dataset used in this study is included in S1 Data in the supporting information file.

## Abbreviations

AIS: Abbreviated Injury Scale
AOR: Adjusted odds ratio
CI: Confidence interval
ED: Emergency department
ISS: Injury Severity Score
