# Supplementary material for: Impact of seat position on survival outcomes and anatomically specific severe injury patterns in four-wheeled motor vehicle accidents: a retrospective cohort study at a community emergency department in Japan
Source: BMC Emerg Med. 2025 Jul 30;25:139. doi: 10.1186/s12873-025-01302-z (PMC12312418; doi:10.1186/s12873-025-01302-z)
Supplement: Supplementary file 2 — Supplementary Material 2: Odds ratios for in-hospital mortality among study participants: front seat occupants versus rear passenger seat occupants. The reference group is the rear passenger seat occupants. The front seat occupants include both the driver seat occupant and front passenger seat occupant. In the multivariable analysis, the front seat position was associated with a higher risk of in-hospital mortality than the rear seat position. aAdjusted for age, sex, admission year, season, presentation time, presentation day, prehospital length of stay, vehicle configuration, collision type, seatbelt use, airbag deployment, and involvement in high-energy trauma. bGood model fit was verified by the Hosmer–Lemeshow test (p = 0.606). The c-statistic for the model was 0.880. OR, odds ratio; CI, confidence interval. [file 12873_2025_1302_MOESM2_ESM.pptx]

## Slide 1
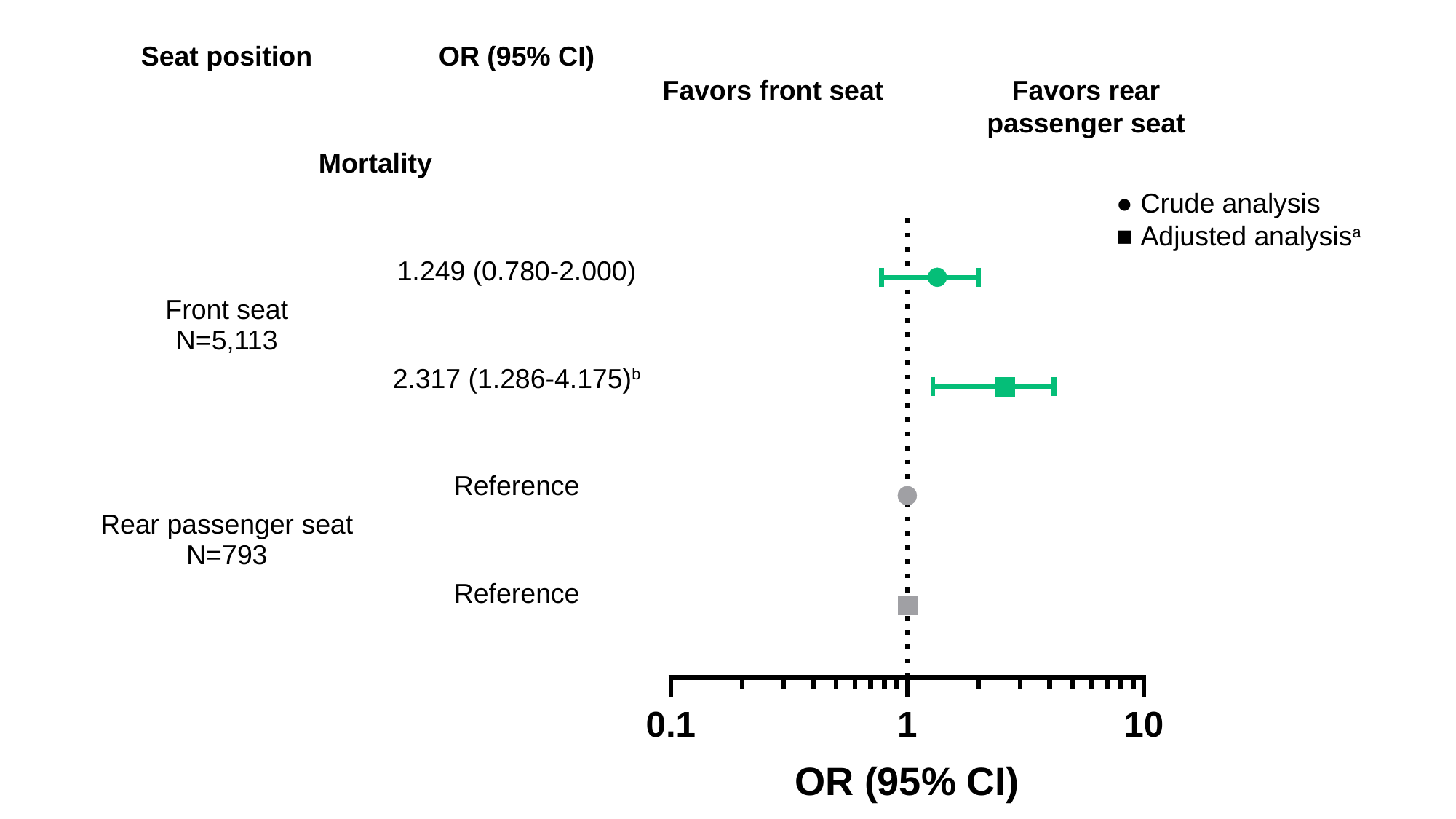

| Seat position | OR (95% CI) |
| --- | --- |
| Mortality | |
| Front seat N=5,113 | 1.249 (0.780-2.000) |
| | 2.317 (1.286-4.175)b |
| Rear passenger seat N=793 | Reference |
| | Reference |
Favors front seat
Favors rear passenger seat
● Crude analysis
■ Adjusted analysisa
