# Supplementary material for: Impact of seat position on survival outcomes and anatomically specific severe injury patterns in four-wheeled motor vehicle accidents: a retrospective cohort study at a community emergency department in Japan
Source: BMC Emerg Med. 2025 Jul 30;25:139. doi: 10.1186/s12873-025-01302-z (PMC12312418; doi:10.1186/s12873-025-01302-z)
Supplement: Supplementary file 3 — Supplementary Material 3: Differences in characteristics between patients who were included in the analysis (complete dataset) and those who were excluded from the analysis because of missing data on traffic accident details. [file 12873_2025_1302_MOESM3_ESM.docx]

**S1 Table. Differences in characteristics between patients who were included in the analysis (complete dataset) and those who were excluded from the analysis because of missing data on traffic accident details**

|  | **Patients who were included in the analysis (n=5,906)** | **Patients who were excluded from analysis due to missing data**  **(n=428)** | ***p*-value** |
| --- | --- | --- | --- |
| **Age (years)** |  |  |  |
| Median (IQR) | 40 (24-58) | 39 (24-57) | 0.494 |
| **Sex** |  |  |  |
| Male | 3037 (51.4) | 191 (44.6) | 0.114 |
| Female | 2869 (48.6) | 237 (55.4) |  |
| **ED admission year** |  |  |  |
| 2000-2007 | 2925 (49.5) | 222 (51.9) | 0.407 |
| 2008-2015 | 1881 (31.8) | 137 (32.0) |  |
| 2016-2022 | 1100 (18.6) | 69 (16.1) |  |
| **Season** |  |  |  |
| Spring (March-May) | 1416 (24.0) | 107 (25.0) | 0.740 |
| Summer (June-August) | 1572 (26.6) | 121 (28.3) |  |
| Autumn (September-November) | 1506 (25.5) | 101 (23.6) |  |
| Winter (December-February) | 1412 (23.9) | 99 (23.1) |  |
| **ED presentation time** |  |  |  |
| 8:00-16:59 | 3125 (52.9) | 143 (33.4) | **<0.001** |
| 17:00-23:59 | 1768 (29.9) | 194 (45.3) |  |
| 0:00-7:59 | 1013 (17.2) | 91 (21.3) |  |
| **ED presentation day** |  |  |  |
| Weekday | 4132 (70.0) | 246 (57.5) | **<0.001** |
| Weekend | 1774 (30.0) | 182 (42.5) |  |
| **Prehospital LOS (min)** |  |  |  |
| Median (IQR) | 42 (29-59) | 40 (28-64) | 0.671 |
| **Vehicle configuration** |  |  |  |
| K-car vehicles | 3352 (56.8) | 252 (58.9) | 0.392 |
| Standard vehicles | 2554 (43.2) | 176 (41.4) |  |
| **High energy trauma** |  |  |  |
| Yes | 2270 (38.4) | 117 (27.3) | **<0.001** |
| No | 3636 (61.6) | 311 (72.7) |  |
| **In-hospital mortality** |  |  |  |
| Yes | 180 (3.0) | 20 (4.7) | 0.063 |
| No | 5726 (97.0) | 408 (95.3) |  |
| **Severe trauma with ISS>15** |  |  |  |
| Yes | 827 (14.0) | 73 (17.1) | 0.081 |
| No | 5079 (86.0) | 355 (82.9) |  |
| **AIS head or neck≥3** |  |  |  |
| Yes | 493 (8.3) | 50 (11.7) | **0.017** |
| No | 5413 (91.7) | 378 (88.3) |  |
| **AIS chest≥3** |  |  |  |
| Yes | 725 (12.3) | 54 (12.6) | 0.836 |
| No | 5181 (87.7) | 374 (87.4) |  |
| **AIS abdomen or pelvic contents**≥**3** |  |  |  |
| Yes | 247 (4.2) | 22 (5.1) | 0.343 |
| No | 5659 (95.8) | 406 (94.9) |  |
| **AIS extremities or pelvic girdle**≥**3** |  |  |  |
| Yes | 401 (6.8) | 23 (5.4) | 0.258 |
| No | 5505 (93.2) | 405 (94.6) |  |

Categorical variables are expressed as n (%), and continuous variables are expressed as median (IQR).

IQR, interquartile range; ED, emergency department; LOS, length of stay; OR, odds ratio; CI, confidence interval; Anatomical-specific severe injuries were defined with AIS≧3 of each body components. AIS, Abbreviated Injury Scale; ISS, Injury Severity Score.
