# Supplementary material for: Impact of seat position on survival outcomes and anatomically specific severe injury patterns in four-wheeled motor vehicle accidents: a retrospective cohort study at a community emergency department in Japan
Source: BMC Emerg Med. 2025 Jul 30;25:139. doi: 10.1186/s12873-025-01302-z (PMC12312418; doi:10.1186/s12873-025-01302-z)
Supplement: Supplementary file 4 — Supplementary Material 4: Odds ratios for in-hospital mortality among (A) male and (B) female subgroups. The reference group is the driver seat occupants. In the female subgroup, the multivariable analysis showed that the rear passenger seat position was associated with a lower risk of mortality than the driver seat position. A similar trend was observed in the male subgroup, although the difference did not reach statistical significance. aAdjusted for age, sex, admission year, season, presentation time, presentation day, prehospital length of stay, vehicle configuration, collision type, seatbelt use, airbag deployment, and involvement in high-energy trauma. bGood model fit was verified by the Hosmer–Lemeshow test (p = 0.493); the c-statistic for the model was 0.857. cGood model fit was verified by the Hosmer–Lemeshow test (p = 0.828); the c-statistic for the model was 0.918. OR, odds ratio; CI, confidence interval. *p < 0.025. [file 12873_2025_1302_MOESM4_ESM.pptx]

## Slide 1
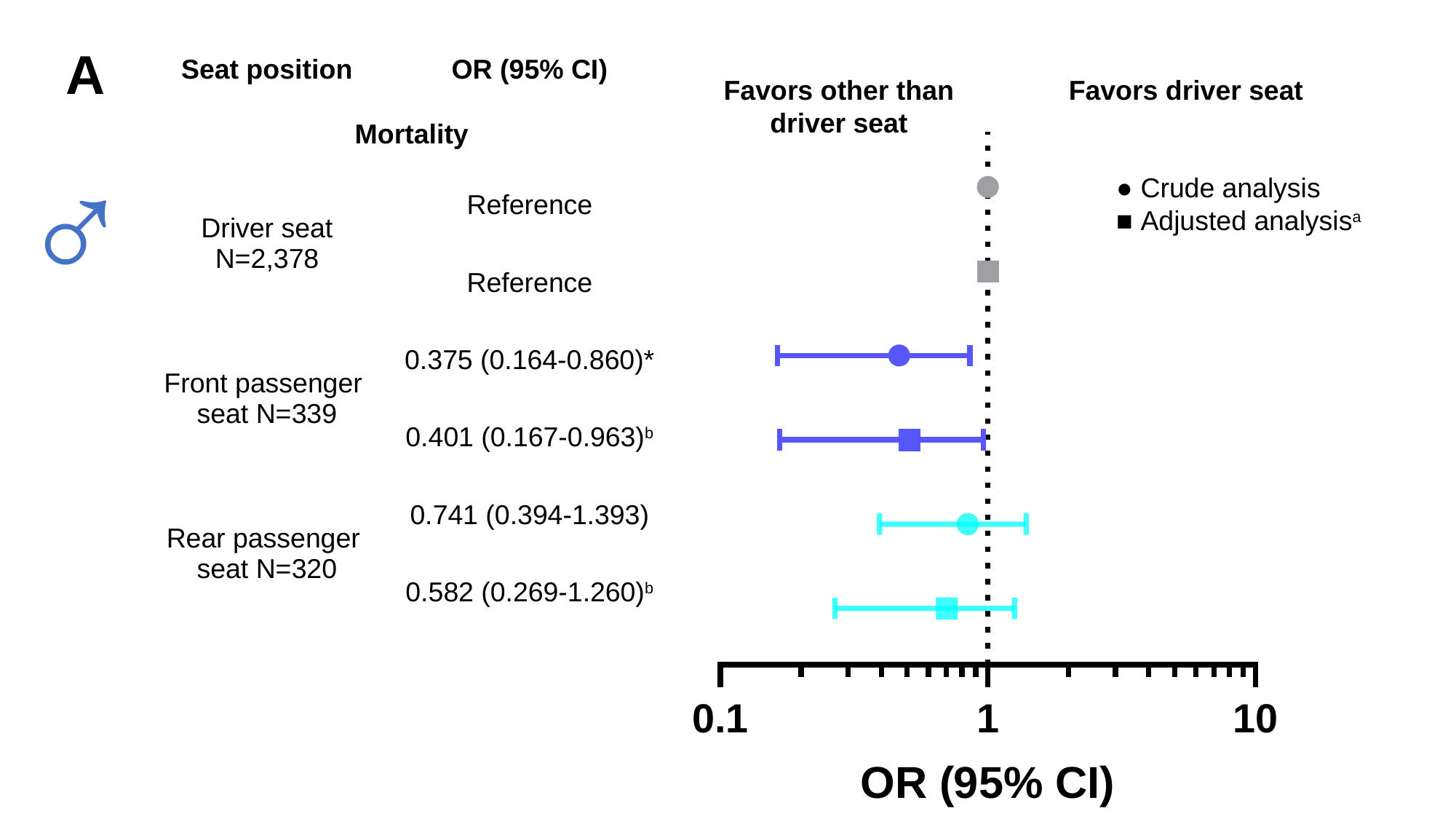

A
| Seat position | OR (95% CI) |
| --- | --- |
| Mortality | |
| Driver seat N=2,378 | Reference |
| | Reference |
| Front passenger seat N=339 | 0.375 (0.164-0.860)\* |
| | 0.401 (0.167-0.963)b |
| Rear passenger seat N=320 | 0.741 (0.394-1.393) |
| | 0.582 (0.269-1.260)b |
Favors driver seat
Favors other than
driver seat
♂
● Crude analysis
■ Adjusted analysisa

## Slide 2
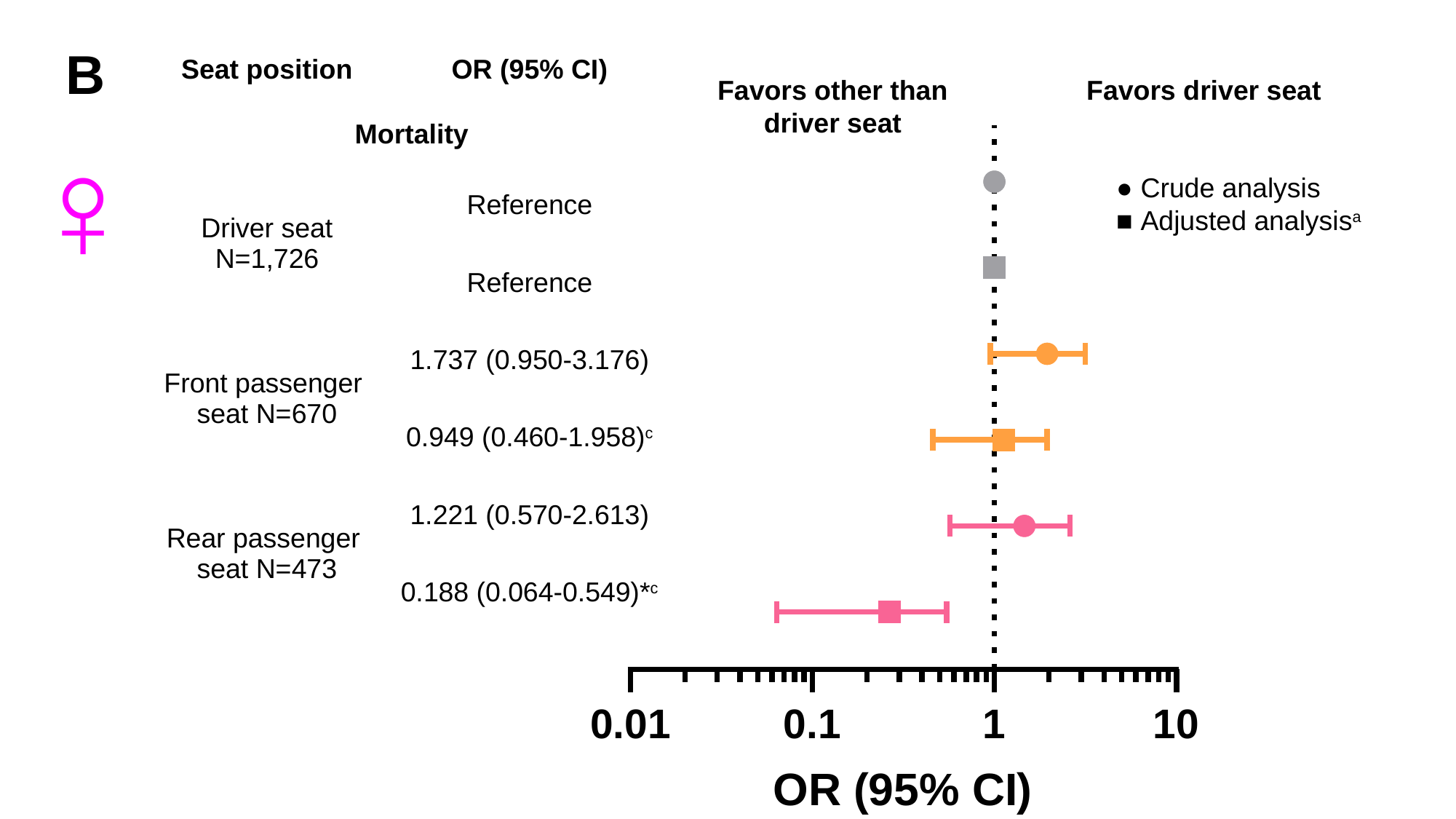

B
| Seat position | OR (95% CI) |
| --- | --- |
| Mortality | |
| Driver seat N=1,726 | Reference |
| | Reference |
| Front passenger seat N=670 | 1.737 (0.950-3.176) |
| | 0.949 (0.460-1.958)c |
| Rear passenger seat N=473 | 1.221 (0.570-2.613) |
| | 0.188 (0.064-0.549)\*c |
Favors driver seat
Favors other than
driver seat
♀
● Crude analysis
■ Adjusted analysisa
