# Supplementary material for: Impact of seat position on survival outcomes and anatomically specific severe injury patterns in four-wheeled motor vehicle accidents: a retrospective cohort study at a community emergency department in Japan
Source: BMC Emerg Med. 2025 Jul 30;25:139. doi: 10.1186/s12873-025-01302-z (PMC12312418; doi:10.1186/s12873-025-01302-z)
Supplement: Supplementary file 5 — Supplementary Material 5: In-hospital mortality among study participants. [file 12873_2025_1302_MOESM5_ESM.docx]

# **S2 Table. In-hospital mortality among study participants**

|  | **Seat position** | | |
| --- | --- | --- | --- |
|  | **Driver seat**  **(n = 4,104)** | **Passenger seat**  **(n = 1,009)** | **Back seat**  **(n = 793)** |
| **In-hospital mortality** |  |  |  |
| Yes | 136 (3.3) | 24 (2.4) | 20 (2.5) |
| No | 3,968 (96.7) | 985 (97.6) | 773 (97.5) |

Data are expressed as n (%).
