# Supplementary material for: Impact of seat position on survival outcomes and anatomically specific severe injury patterns in four-wheeled motor vehicle accidents: a retrospective cohort study at a community emergency department in Japan
Source: BMC Emerg Med. 2025 Jul 30;25:139. doi: 10.1186/s12873-025-01302-z (PMC12312418; doi:10.1186/s12873-025-01302-z)
Supplement: Supplementary file 6 — Supplementary Material 6: Odds ratios of severe trauma (ISS of > 15) and anatomical site-specific severe injury (AIS score of ≥ 3) for each body component among (A) male and (B) female subgroups. The reference group is the driver seat occupants. An adjusted association of decreased risk of severe trauma (ISS of > 15) and severe chest injuries was observed regardless of sex differences. aAdjusted for age, sex, admission year, season, presentation time, presentation day, prehospital length of stay, vehicle configuration, collision type, seatbelt use, airbag deployment, and involvement in high-energy trauma. bGood model fit was verified by the Hosmer–Lemeshow test (p = 0.365); the c-statistic for the model was 0.795. cGood model fit was verified by the Hosmer–Lemeshow test (p = 0.361); the c-statistic for the model was 0.888. dGood model fit was verified by the Hosmer–Lemeshow test (p = 0.661); the c-statistic for the model was 0.841. eGood model fit was verified by the Hosmer–Lemeshow test (p = 0.478); the c-statistic for the model was 0.855. fGood model fit was verified by the Hosmer–Lemeshow test (p = 0.455); the c-statistic for the model was 0.838. gThe Hosmer–Lemeshow test p-value was < 0.001; the c-statistic for the model was 0.871. hGood model fit was verified by the Hosmer–Lemeshow test (p = 0.395); the c-statistic for the model was 0.818. iGood model fit was verified by the Hosmer–Lemeshow test (p = 0.763); the c-statistic for the model was 0.861. jGood model fit was verified by the Hosmer–Lemeshow test (p = 0.742); the c-statistic for the model was 0.851. kGood model fit was verified by the Hosmer–Lemeshow test (p = 0.785). AIS, Abbreviated Injury Scale; ISS, Injury Severity Score; OR, odds ratio; CI, confidence interval. *p < 0.025. [file 12873_2025_1302_MOESM6_ESM.pptx]

## Slide 1
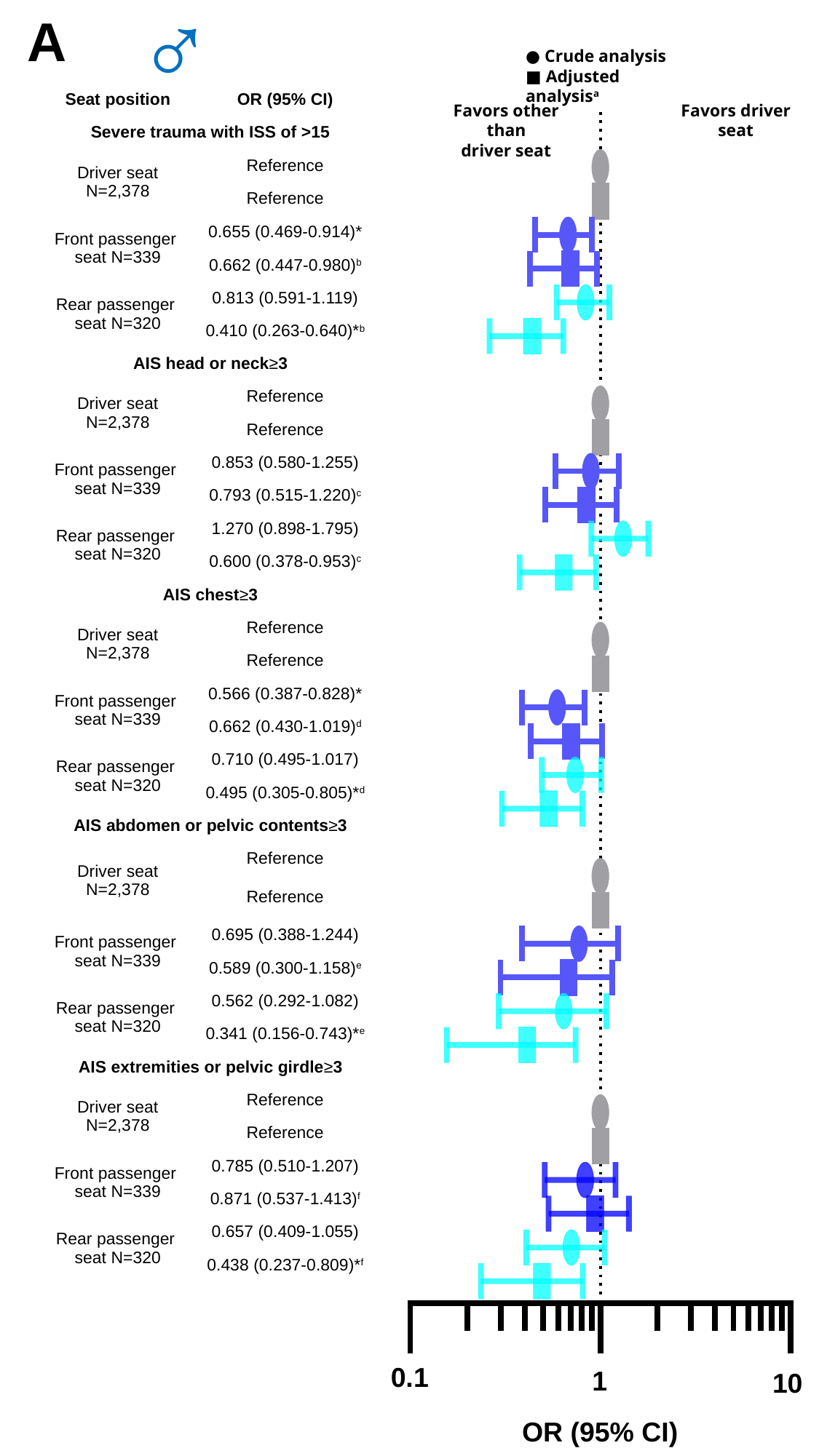

♂
A
● Crude analysis
■ Adjusted analysisa
| Seat position | OR (95% CI) |
| --- | --- |
| Severe trauma with ISS of >15 | |
| Driver seat N=2,378 | Reference |
| | Reference |
| Front passenger seat N=339 | 0.655 (0.469-0.914)\* |
| | 0.662 (0.447-0.980)b |
| Rear passenger seat N=320 | 0.813 (0.591-1.119) |
| | 0.410 (0.263-0.640)\*b |
| AIS head or neck≥3 | |
| Driver seat N=2,378 | Reference |
| | Reference |
| Front passenger seat N=339 | 0.853 (0.580-1.255) |
| | 0.793 (0.515-1.220)c |
| Rear passenger seat N=320 | 1.270 (0.898-1.795) |
| | 0.600 (0.378-0.953)c |
| AIS chest≥3 | |
| Driver seat N=2,378 | Reference |
| | Reference |
| Front passenger seat N=339 | 0.566 (0.387-0.828)\* |
| | 0.662 (0.430-1.019)d |
| Rear passenger seat N=320 | 0.710 (0.495-1.017) |
| | 0.495 (0.305-0.805)\*d |
| AIS abdomen or pelvic contents≥3 | |
| Driver seat N=2,378 | Reference |
| | Reference |
| Front passenger seat N=339 | 0.695 (0.388-1.244) |
| | 0.589 (0.300-1.158)e |
| Rear passenger seat N=320 | 0.562 (0.292-1.082) |
| | 0.341 (0.156-0.743)\*e |
| AIS extremities or pelvic girdle≥3 | |
| Driver seat N=2,378 | Reference |
| | Reference |
| Front passenger seat N=339 | 0.785 (0.510-1.207) |
| | 0.871 (0.537-1.413)f |
| Rear passenger seat N=320 | 0.657 (0.409-1.055) |
| | 0.438 (0.237-0.809)\*f |
Favors other than
driver seat
Favors driver seat
0.1
1
10
OR (95% CI)

## Slide 2
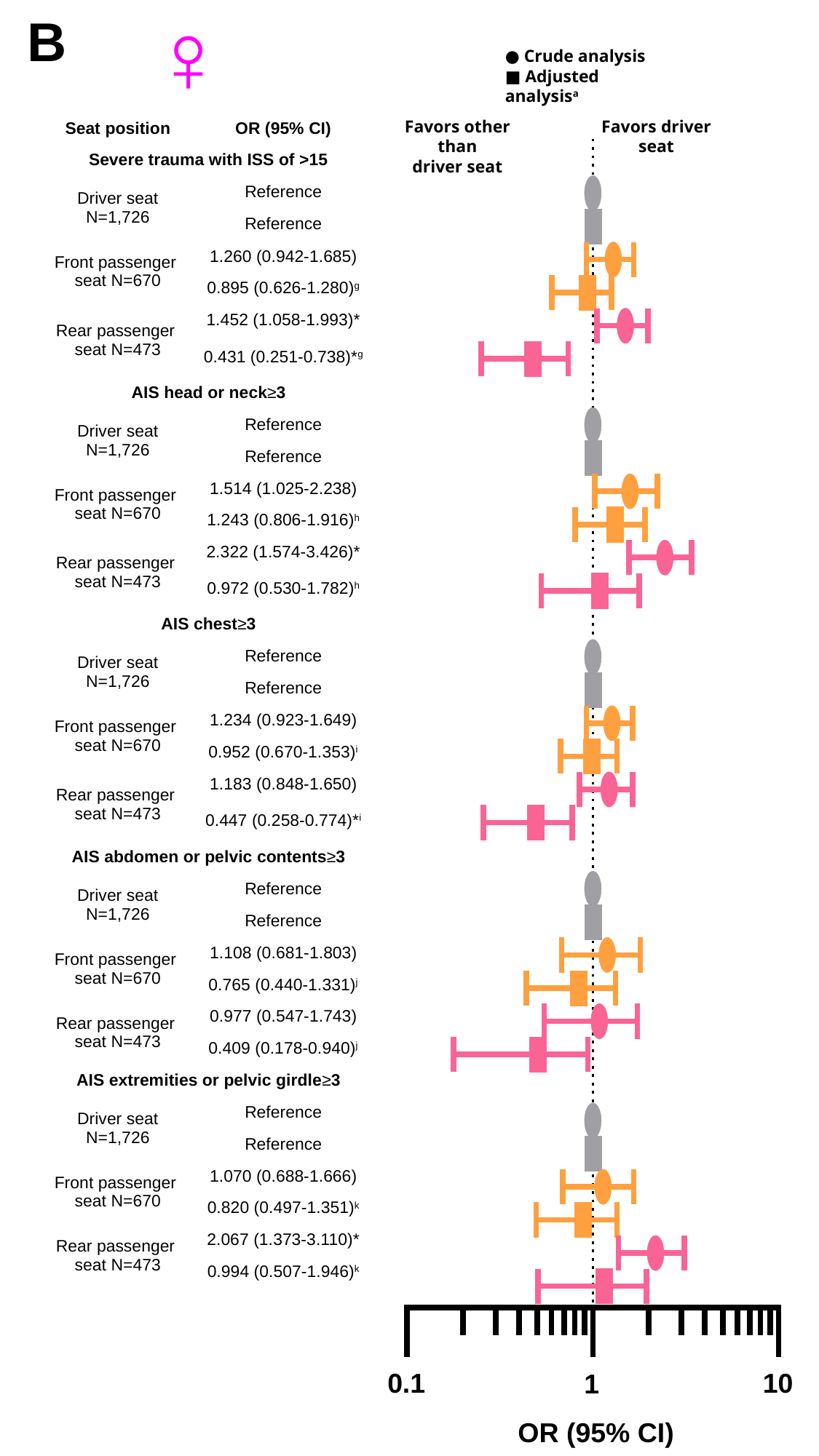

♀
B
● Crude analysis
■ Adjusted analysisa
Favors other than
driver seat
Favors driver seat
| Seat position | OR (95% CI) |
| --- | --- |
| Severe trauma with ISS of >15 | |
| Driver seat N=1,726 | Reference |
| | Reference |
| Front passenger seat N=670 | 1.260 (0.942-1.685) |
| | 0.895 (0.626-1.280)g |
| Rear passenger seat N=473 | 1.452 (1.058-1.993)\* |
| | 0.431 (0.251-0.738)\*g |
| AIS head or neck≥3 | |
| Driver seat N=1,726 | Reference |
| | Reference |
| Front passenger seat N=670 | 1.514 (1.025-2.238) |
| | 1.243 (0.806-1.916)h |
| Rear passenger seat N=473 | 2.322 (1.574-3.426)\* |
| | 0.972 (0.530-1.782)h |
| AIS chest≥3 | |
| Driver seat N=1,726 | Reference |
| | Reference |
| Front passenger seat N=670 | 1.234 (0.923-1.649) |
| | 0.952 (0.670-1.353)i |
| Rear passenger seat N=473 | 1.183 (0.848-1.650) |
| | 0.447 (0.258-0.774)\*i |
| AIS abdomen or pelvic contents≥3 | |
| Driver seat N=1,726 | Reference |
| | Reference |
| Front passenger seat N=670 | 1.108 (0.681-1.803) |
| | 0.765 (0.440-1.331)j |
| Rear passenger seat N=473 | 0.977 (0.547-1.743) |
| | 0.409 (0.178-0.940)j |
| AIS extremities or pelvic girdle≥3 | |
| Driver seat N=1,726 | Reference |
| | Reference |
| Front passenger seat N=670 | 1.070 (0.688-1.666) |
| | 0.820 (0.497-1.351)k |
| Rear passenger seat N=473 | 2.067 (1.373-3.110)\* |
| | 0.994 (0.507-1.946)k |
0.1
10
1
OR (95% CI)
