# Supplementary material for: Impact of seat position on survival outcomes and anatomically specific severe injury patterns in four-wheeled motor vehicle accidents: a retrospective cohort study at a community emergency department in Japan
Source: BMC Emerg Med. 2025 Jul 30;25:139. doi: 10.1186/s12873-025-01302-z (PMC12312418; doi:10.1186/s12873-025-01302-z)
Supplement: Supplementary file 7 — Supplementary Material 7: Severe trauma with ISS of > 15 and anatomical site-specific severe injury with AIS score of ≥ 3 for each body component among study participants. [file 12873_2025_1302_MOESM7_ESM.docx]

# **S3 Table. Severe trauma with ISS of >15 and anatomical site-specific severe injury with AIS score of ≥3 for each body component among study participants**

|  | **Seat position** | | |
| --- | --- | --- | --- |
|  | **Driver seat**  **(n = 4,104)** | **Front passenger seat**  **(n = 1,009)** | **Rear passenger seat**  **(n = 793)** |
| **Severe trauma with ISS of >15** |  |  |  |
| Yes | 598 (14.6) | 119 (11.8) | 110 (13.9) |
| No | 3,506 (85.4) | 890 (88.2) | 683 (86.1) |
| **AIS score for head or neck ≥ 3** |  |  |  |
| Yes | 332 (8.1) | 74 (7.3) | 87 (11.0) |
| No | 3,772 (91.9) | 935 (92.7) | 706 (89.0) |
| **AIS score for chest ≥ 3** |  |  |  |
| Yes | 530 (12.9) | 107 (10.6) | 88 (11.1) |
| No | 3,574 (87.1) | 902 (89.4) | 705 (88.9) |
| **AIS score for abdominal or pelvic contents ≥ 3** |  |  |  |
| Yes | 185 (4.5) | 37 (3.7) | 25 (3.2) |
| No | 3,919 (95.5) | 972 (96.3) | 768 (96.8) |
| **AIS score for extremities or pelvic girdle ≥ 3** |  |  |  |
| Yes | 289 (7.0) | 54 (5.4) | 58 (7.3) |
| No | 3,815 (93.0) | 955 (94.6) | 735 (92.7) |

Data are expressed as n (%). AIS, Abbreviated Injury Scale. ISS, Injury Severity Score.
