# Supplementary material for: Impact of seat position on survival outcomes and anatomically specific severe injury patterns in four-wheeled motor vehicle accidents: a retrospective cohort study at a community emergency department in Japan
Source: BMC Emerg Med. 2025 Jul 30;25:139. doi: 10.1186/s12873-025-01302-z (PMC12312418; doi:10.1186/s12873-025-01302-z)
Supplement: Supplementary file 8 — Supplementary Material 8: Factors associated with in-hospital mortality among rear passenger seat occupants. [file 12873_2025_1302_MOESM8_ESM.docx]

**S4 Table. Factors associated with in-hospital mortality among rear passenger seat occupants**

|  | **Mortality** | | **Crude analysis** | |
| --- | --- | --- | --- | --- |
|  | **Survival**  **(n=773)** | **Death**  **(n=20)** | **OR (95% CI)** | ***p*-value** |
| **Age (years)** |  |  |  |  |
| Median (IQR) | 25 (10-55) | 62 (18.5-76) | 1.026 (1.009-1.043) | **0.003** |
| **Sex** |  |  |  |  |
| Male | 309 (96.6) | 11 (3.4) | 1.835 (0.752-4.481) | 0.182 |
| Female | 464 (98.1) | 9 (1.9) | Reference |  |
| **ED admission year** |  |  |  |  |
| 2000-2007 | 399 (97.6) | 10 (2.4) | Reference |  |
| 2008-2015 | 237 (96.7) | 8 (3.3) | 1.347 (0.524-3.460) | 0.536 |
| 2016-2022 | 137 (98.6) | 2 (1.4) | 0.582 (0.126-2.691) | 0.489 |
| **Season** |  |  |  |  |
| Spring (March-May) | 175 (97.2) | 5 (2.8) | Reference |  |
| Summer (June-August) | 205 (96.2) | 8 (3.8) | 1.366 (0.439-4.251) | 0.590 |
| Autumn (September-November) | 188 (97.4) | 5 (2.6) | 0.931 (0.265-3.270) | 0.911 |
| Winter (December-February) | 205 (99.0) | 2 (1.0) | 0.341 (0.065-1.782) | 0.202 |
| **ED presentation time** |  |  |  |  |
| 8:00-16:59 | 417 (97.7) | 10 (2.3) | Reference |  |
| 17:00-23:59 | 238 (97.9) | 5 (2.1) | 0.876 (0.296-2.593) | 0.811 |
| 0:00-7:59 | 118 (95.9) | 5 (4.1) | 1.767 (0.592-5.270) | 0.307 |
| **ED presentation day** |  |  |  |  |
| Weekday | 461 (97.3) | 13 (2.7) | Reference |  |
| Weekend | 312 (97.8) | 7 (2.2) | 0.796 (0.314-2.017) | 0.630 |
| **Prehospital length of stay (min)** |  |  |  |  |
| Median (IQR) | 45 (29-65.5) | 60.5 (47-75.5) | 1.015 (1.001-1.030) | **0.032** |
| **Vehicle configuration** |  |  |  |  |
| K-car vehicles | 202 (98.5) | 3 (1.5) | 0.499 (0.145-1.720) | 0.271 |
| Standard vehicles | 571 (97.1) | 17 (2.9) | Reference |  |
| **Collision type** |  |  |  |  |
| Frontal collision | 305 (97.1) | 9 (2.9) | Reference |  |
| Lateral collision | 153 (98.7) | 2 (1.3) | 0.581 (0.124-2.719) | 0.490 |
| Rear-end collision | 95 (100) | 0 (0) | N/A |  |
| Rollover collision | 92 (98.9) | 1 (1.1) | 0.483 (0.060-3.861) | 0.493 |
| Complex collision | 97 (93.3) | 7 (6.7) | 3.207 (1.165-8.827) | **0.024** |
| Other type of collision | 31 (96.9) | 1 (3.1) | 1.434 (0.176-11.685) | 0.736 |
| **Seat belt** |  |  |  |  |
| Unbelted | 611 (97.3) | 17 (2.7) | Reference |  |
| Belted | 132 (100) | 0 (0) | N/A |  |
| Improper seatbelt use | 30 (90.9) | 3 (9.1) | 4.371 (1.215-15.727) | **0.024** |
| **Airbag** |  |  |  |  |
| Not equipped | 765 (97.5) | 20 (2.5) | Reference |  |
| Equipped and non-deployment | 4 (100) | 0 (0) | N/A |  |
| Equipped and deployment | 4 (100) | 0 (0) | N/A |  |
| **High energy trauma** |  |  |  |  |
| Yes | 265 (93.6) | 18 (6.4) | 17.253 (3.973-74.917) | **<0.001** |
| No | 508 (99.6) | 2 (0.4) | Reference |  |

Categorical variables are expressed as n (%), and continuous variables are expressed as median (IQR). IQR, interquartile range; ED, emergency department; OR, odds ratio; CI, confidence interval.
